# Epidermal Remodeling in *Caenorhabditis elegans* Dauers Requires the Nidogen Domain Protein DEX-1

**DOI:** 10.1534/genetics.118.301557

**Published:** 2018-11-01

**Authors:** Kristen M. Flatt, Caroline Beshers, Cagla Unal, Jennifer D. Cohen, Meera V. Sundaram, Nathan E. Schroeder

**Affiliations:** * Program in Neuroscience, University of Illinois at Urbana-Champaign, Illinois 61801-4730; † Department of Genetics, University of Pennsylvania Perelman School of Medicine, Philadelphia, Pennsylvania 19104-6145; ‡ Department of Crop Sciences, University of Illinois at Urbana-Champaign, Illinois 61801-4730

**Keywords:** polyphenism, cuticlin, SNED1, tectorin, DYF-7, extracellular matrix

## Abstract

Phenotypic plasticity is a critical component of an organism’s ability to thrive in a changing environment. The free-living nematode *Caenorhabditis elegans* adapts to unfavorable environmental conditions by pausing reproductive development and entering a stress-resistant larval stage known as dauer. The transition into dauer is marked by vast morphological changes, including remodeling of epidermis, neurons, and muscle. Although many of these dauer-specific traits have been described, the molecular basis of dauer-specific remodeling is still poorly understood. Here we show that the nidogen domain-containing protein DEX-1 facilitates stage-specific tissue remodeling during dauer morphogenesis. DEX-1 was previously shown to regulate sensory dendrite formation during embryogenesis. We find that DEX-1 is also required for proper remodeling of the stem cell-like epidermal seam cells. *dex-1* mutant dauers lack distinct lateral cuticular alae during dauer and have increased sensitivity to sodium dodecyl sulfate. Furthermore, we find that DEX-1 is required for proper dauer mobility. We show that DEX-1 is secreted from the seam cells during dauer, but acts locally in a cell-autonomous manner. We find that *dex-1* expression during dauer is regulated through DAF-16/FOXO–mediated transcriptional activation. Finally, we show that *dex-1* acts with a family of zona pellucida domain-encoding genes to regulate dauer-specific epidermal remodeling. Taken together, our data indicate that DEX-1 is an extracellular matrix component that plays a central role in *C. elegans* epidermal remodeling during dauer.

TO survive changing environments, organisms modify their phenotype (*i.e*., phenotypic plasticity). Tissue remodeling is an important component of stress-induced phenotypic plasticity. For example, desert locusts are capable of altering their morphology between distinct “gregarious” and “solitarious” phases based on population density (Pener and Simpson 2009), and certain species of butterfly change their body morphology and wing color based on seasonal cues (Windig 1994). While these large-scale displays of phenotypic plasticity are readily observed, the molecular basis of tissue remodeling in response to environmental inputs is often unclear.

*Caenorhabditis elegans* is a useful animal to investigate the molecular mechanisms that facilitate stress-induced remodeling. Under favorable growth conditions, *C. elegans* develops continuously through four larval stages (L1–L4), into a reproductive adult. However, under unfavorable environmental conditions, *C. elegans* larvae arrest their development at the second larval molt and enter the stress-resistant dauer stage (Cassada and Russell 1975; Golden and Riddle 1984). Dauers are specialized, nonfeeding larvae capable of withstanding extended periods of adverse environmental conditions. Dauer-specific stress resistance is likely facilitated by several morphological changes that occur during dauer formation. For example, dauers have both structural and biochemical differences in their epidermis and cuticle compared with nondauers (Cox *et al.* 1981; Blaxter 1993). Two morphological features characteristic of dauer formation are a general radial shrinkage of the body and the formation of longitudinal cuticular ridges called alae (Figure 1A).

**Figure 1:**
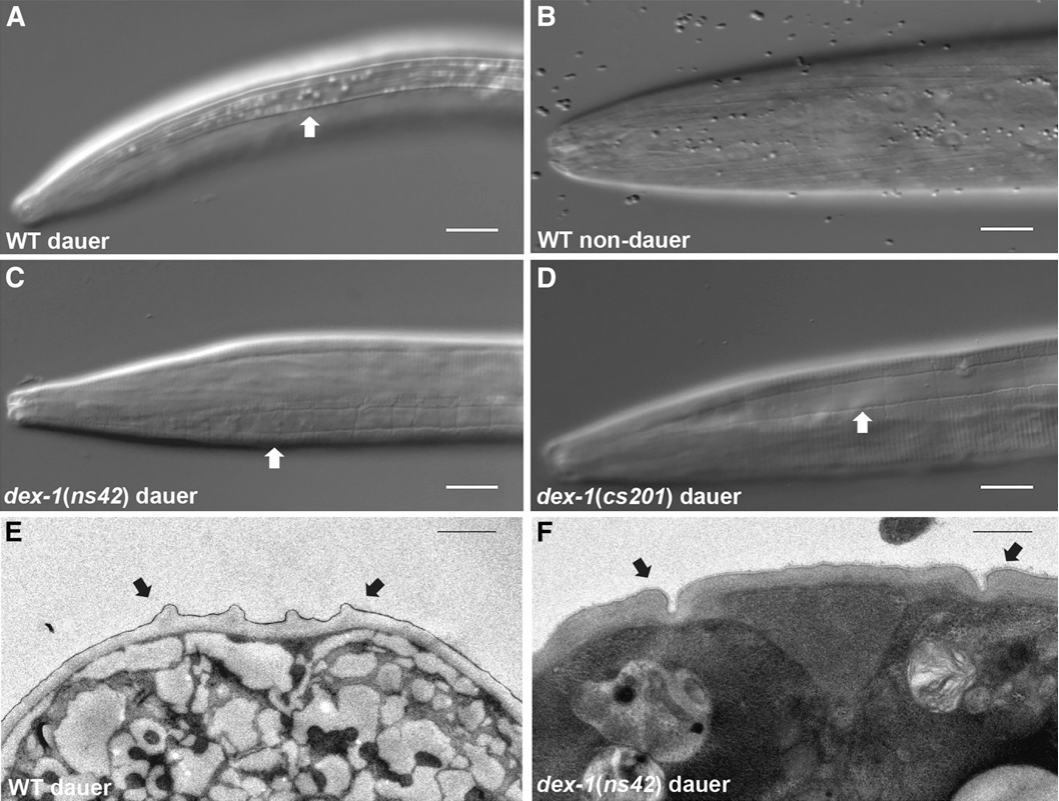
*dex-1* mutants have defects in lateral alae formation. (A) Wild-type dauers have prominent lateral alae (arrows) that are not present in the comparable nondauer L3 stage (B). (C) *dex-1(ns42*) and (D) *dex-1(cs201*) mutant dauer alae are indistinct. All animals are lying laterally. Bar, 10 μm. (E and F) Transmission electron micrograph showing lateral alae (arrows) of a wild-type dauer (E) and *dex-1(ns42)* mutant dauer (F). Bar, 1 μm.

Radial shrinkage and alae formation are regulated by a set of lateral hypodermal seam cells (Singh and Sulston 1978; Melendez *et al.* 2003). Seam cell function and remodeling are critical for proper dauer morphology and increased environmental resistance. The seam cells also have stem cell-like properties; during nondauer development, the seam cells undergo asymmetrical divisions at larval molts to produce an anterior differentiated cell and a posterior seam cell (Sulston and Horvitz 1977). Alternatively, if the animal enters dauer diapause, the seam cells shrink and stop dividing (Melendez *et al.* 2003; Karp and Ambros 2012)

The decision to enter dauer typically results in an all or none phenotype where dauers have every dauer morphological characteristic. However, mutations in some genes necessary for dauer formation result in an intermediate dauer phenotype, called partial dauers, in which the animals will display some, but not all, dauer characteristics (Albert and Riddle 1988). Here, we characterize the role of DEX-1, a protein similar to mammalian tectorin and SNED1, in seam cell remodeling during dauer formation. DEX-1 is a transmembrane protein with two extracellular nidogen domains, and is required for proper sensory neuron dendrite formation during embryogenesis (Heiman and Shaham 2009). We find that the *dex-1* mutant dauers have alae and cuticle defects that are not suppressed by dauer-constitutive (*daf-c*) mutants. Furthermore, we show that DEX-1 can function as a secreted protein and localizes in or near dauer alae. Finally, we find that *dex-1* is upregulated in seam cells during dauer in a DAF-16–dependent manner. DEX-1 was previously shown to function with a zona pellucida (ZP) domain-containing protein, DYF-7, to mediate dendrite extension (Heiman and Shaham 2009). Our data suggest that DEX-1 acts along with additional ZP-domain proteins to regulate seam cell remodeling. Combined with previous data demonstrating a role for DEX-1 in sensory dendrite adhesion (Heiman and Shaham 2009), and data in the accompanying article showing roles for DEX-1 in epithelial shaping in the embryo (Cohen *et al.* 2018), our data suggest that DEX-1 is an extracellular matrix (ECM) component that plays a role in modulating cell shape of several cell types throughout development.

## Materials and Methods

### Strains and plasmids

All strains were grown under standard conditions unless otherwise noted (Brenner 1974). The wild-type Bristol N2 strain and the following mutant strains were used: DR27 *daf-16(m27)* I, FX01126 *cut-1(tm1126)* II, CHB27 *dex-1(ns42)* III, UP2571 *dex-1(cs201)*III; *csEx402[dex-1p*::*dex-1a* + *unc-199p*::*gfp]*, CB1372 *daf-7(e1372)* III, DR129 *daf-2(e1370) unc-32(e189)* III, RB1574 *cut-6(ok1919)* III, RB1629 *cut-5(ok2005)* X, SP1735 *dyf-7(m537)* X. All mutant strains were backcrossed at least twice. *dex-1(cs201)* was generated using standard EMS mutagenesis protocols (Brenner 1974; Flibotte *et al.* 2010) and identified based on balancer mapping and whole genome sequencing (Cohen *et al.* 2018). *dex-1(ns42)* was a gift from Dr. Maxwell Heiman (Department of Genetics, Harvard University, Boston, MA). *cut-1(tm1126)* was provided by the Mitani Consortium (Department of Physiology, Tokyo Women’s Medical University School of Medicine, Japan). The following transgenic strains were used: ST65 *ncIs13[ajm-1*::*gfp]* was used to observe the apical junctions of the seam cells (Köppen *et al.* 2001). The IL2 neurons were observed using PT2660 *myIs13[klp-6p*::*gfp+pBx]* III; PT2762 *myIs14[klp-6p*::*gfp+pBx]* V and JK2868 *qIs56*[*lag-2p*::*gfp*] V (Blelloch *et al.* 1999; Ouellet *et al.* 2008; Schroeder *et al.* 2013). The deirid neurons were observed using TG2435 *vtIs1[dat-1p*::*gfp*+*rol-6(su1006*)] V (Nass *et al.* 2002).

The following plasmids were a generous gift from Dr. Maxwell Heiman: pMH7 *dex-1p*::*dex-1*, pMH8 *pha-4p*::*dex-1*, pMH111 *dex-1p(5.7 kb)*::*gfp*, pMH125 *dex-1p(2.1 kb)*::*gfp* (Heiman and Shaham 2009). The *sur-5*::*gfp* construct used for mosaic analysis was a generous gift from Dr. Trent Gu (Gu *et al.* 1998; Yochem *et al.* 1998). Plasmids were constructed using Gibson Assembly (E2611S; New England Biolabs, Beverly, MA) and restriction enzyme cloning (for a complete list of primers and plasmids, see Supplemental Material, Table S1). The seam cell-specific expression plasmid was built by replacing the *dex-1* promoter from pMH7 with a 1.21 kb *cut-5* promoter region. The hypodermal-specific *dex-1* plasmid was constructed by replacing the *dex-1* promoter in pMH7 with the *cut-6* promoter (Muriel *et al.* 2003; Sapio *et al.* 2005). The insulin response sequence was deleted from pMH111 using the Q5 Site Directed Mutagenesis Kit (E05525; New England Biolabs). Additionally, translational reporters *dex-1p*::*sfgfp*::*dex-1* (pJC24) and *dex-1p*::*dex-1(ecto)*::*sfgfp* (pJC15) contain the 2.1 kb *dex-1* promoter and *dex-1* (isoform a) complementary (cDNA) from pMH7 [see Cohen *et al.* (2018)]. *sfgfp* was inserted either at an internal endogenous *Bgl*II restriction site to generate a full-length fusion tagged upstream of the first nidogen domain, or inserted at the 3′ end of a cDNA truncated before the transmembrane domain. See accompanying article (Cohen *et al.* 2018) for further cloning details.

Animals containing extrachromosomal arrays were generated using standard microinjection techniques (Mello *et al.* 1991), and genotypes confirmed using PCR analysis and observation of co-injection markers. *dex-1(ns42)* animals were injected with 20 ng/μl of plasmid and 80 ng/μl of *unc-122p*::*gfp*, *unc-122p*::*rfp* or *sur-5*::*gfp* as the co-injection marker. *dex-1(cs201)* animals were injected with 30 ng/μl of pJC15 or pJC24 and 50 ng/μl of co-injection marker pHS4 (*lin-48p*::*mrfp*).

Domain schematics were constructed using the wormweb.org Exon-Intron Graphic Maker. Domain locations were determined using the Simple Modular Architecture Research Tool domain prediction software (Schultz *et al.* 1998). The additional low complexity area with similarity to zonadhesin was assigned based on previous work (Heiman and Shaham 2009).

### Dauer formation assays

Dauers were induced by one of two methods. For nontemperature-sensitive strains, we used plates containing crude dauer pheromone extracted by previously established procedures (Vowels and Thomas 1992; Schroeder and Flatt 2014). Dauers were induced in *daf-16(m27)* mutants using plates with high pheromone concentrations (EC_90_) (Gottlieb and Ruvkun 1994; Schroeder and Flatt 2014). For temperature-sensitive strains with mutations in *daf-7(e1372)* or *daf-2(e1370)*, dauers were induced using the restrictive temperature of 25° (Riddle *et al.* 1981).

### Microscopy and rescue analysis

Unless otherwise specified, animals were mounted onto 4% agarose pads and immobilized with 0.1 or 0.01 M levamisole for dauers and nondauer or partial dauers, respectively. In our hands, dauers frequently lay in a dorsal-ventral position following anesthesia. Therefore, to image the lateral side, dauers were immobilized by mounting on 4% agarose pads with Polybead Polystyrene 0.10 μm microspheres (Polysciences Inc., #00876) (Kim *et al.* 2013). A Zeiss AxioImager microscope equipped with DIC and fluorescence optics was used to collect images. For radial constriction experiments, Z-stack images were taken and Z-projections were made using FIJI. Diameter measurements were taken near the center of the terminal pharyngeal bulb. Measurement data were analyzed using a one-way ANOVA with Bonferroni’s multiple comparisons test using GraphPad Prism 6 software. The resulting Z-projections were used to measure body diameter. For seam cell area analysis, area was measured for V2pap, V2ppp, and V3pap and averaged to give one measurement per animal (Sulston and Horvitz 1977). Seam cell measurement data were analyzed by an unpaired *t*-test. For confocal microscopy of the IL2 neurons, dauers were mounted on 10% agarose pads and anesthetized with 0.1 or 0.01 M levamisole. Animals were imaged using a Zeiss LSM 880 confocal microscope.

For transmission electron microscopy of *dex-1(ns42)* and N2 animals, dauer larvae were induced using pheromone plates and processed for high-pressure freezing and freeze substitution modified from previously established methods (Hall *et al.* 2012; Manning and Richmond 2015). Using OP50 *Escherichia coli* as a substrate and 1% propylene phenoxetol in M9 buffer as an anesthetic, animals were loaded into a metal specimen carrier coated with 1-hexadecane and frozen in an HPM 010 high-pressure freezer. Freeze substitution was performed in an FS-8500 freeze substitution system using 2% OsO_4_ (Electron Microscopy Sciences), 0.1% uranyl acetate (Polysciences) in 2% H_2_0, and 100% acetone. Samples were held at −90° for 110 hr, then warmed to −20° at the rate of 5° per hour (14 hr). Samples were then held at −20° for 16 hr, then warmed to 0° at the rate of 5° per hour (4 hr). Samples were washed three times in prechilled (0°) 100% acetone and incubated at 0° for 1 hr after the final wash. Samples were then warmed to room temperature and washed an additional two times with 100% acetone. Samples were infiltrated with 1:1 Polybed812 (Polysciences) resin:acetone for 6 hr, 2:1 resin:acetone for 14 hr, and 100% resin for 72 hr. All infiltration steps were incubated on an orbital shaker at room temperature. Samples were then embedded in molds in 100% resin plus DMP-30 hardener (Polysciences) and baked at 60° for 48 hr, then 70 nm sections were cut with a diamond knife using a PowerTome PC ultramicrotome and collected onto formvar-coated copper slot grids. Samples were imaged with a Philips CM200 transmission electron microscope.

### Sodium dodecyl sulfate sensitivity assays

Sodium dodecyl sulfate (SDS) dose-response assays were performed using 12-well culture dishes containing M9 buffer and specified concentrations of SDS. Dauers were exposed to SDS for 30 min and scored as alive if movement was observed following stimulation with an eyelash. Each concentration was tested in triplicate with each experiment containing a separate wild-type (N2) control. Dose-response curves and LD_50_ values were determined by testing 20 dauers per treatment at each concentration, with three independent experiments. The LD_50_ and 95% confidence interval of each concentration was calculated using probit analysis in Minitab 18. LD_50_ values were considered significantly different if the 95% confidence intervals did not overlap. Significant difference was denoted with a single asterisk. Single concentration assays were conducted at 0.1% SDS with 20 dauers for each genotype and three independent experiments. Data were analyzed using a one proportion exact method analysis in Minitab 18 and considered significantly different if the 95% confidence intervals did not overlap. Significant difference was denoted with a single asterisk.

### Fluorescent bead and behavioral assays

Fluorescent bead assays were carried out using established methods (Nika *et al.* 2016). Fluorescent beads (L3280; Sigma) were added to a ×10 concentrated OP50 *E. coli* overnight culture. Fresh NGM plates were seeded with 65 μl of the bead/bacteria suspension and allowed to dry. Twenty nematodes were added to the plate and incubated at 20° for 40 min. Worms were observed for the presence of fluorescent beads in the intestinal tract. The experiment was performed twice.

Pharyngeal pumping assays were modified from previous established methods (Keane and Avery 2003). *dex-1(ns42)* and wild-type dauers were transferred to seeded NGM plates and allowed to recover for 10 min. After recovery, each animal was scored individually for 2 min, and then removed from the plate. Data were analyzed by an unpaired *t*-test, using GraphPad Prism 6 software.

For movement assays, animals were transferred to unseeded NGM plates and allowed to sit at room temperature for 10 min before being assayed. Animals were stimulated near the anus with an eyelash and the number of body bends was scored. Counting was stopped if the animal did not complete another body bend within 5 sec of stopping, or if the animal reversed direction. Each animal was scored twice and then removed from the plate. Counts were averaged and then analyzed using a nonparametric Kruskal–Wallis test, with Dunn’s multiple comparisons test for dauers and the Mann–Whitney *U* test for adult animals, using GraphPad Prism 6 software.

### DEX-1 expression analysis

To analyze *dex-1p*::*gfp* expression in the seam, images of dauer animals were taken using identical fluorescence settings and exposure times (10 ms). The fluorescence intensities of the V2pap, V2ppp, and V3pap seam cells were measured using established methods (McCloy *et al.* 2014). Each cell was outlined and the area, integrated density, and mean gray value were measured. Measurements were also taken for areas without fluorescence surrounding the cell. The total corrected cell fluorescence [TCCF = integrated density − (area of selected cell × mean fluorescence of background reading)] was calculated for each cell. The intensities of the three cells from each worm were averaged such that each nematode comprised a single data point. Variability in copy number between *dex-1p*::*gfp* and *daf-16(m27)*; *dex-1p*::*gfp* was controlled by using the same transgene in both wild-type and *daf-16(m27)* backgrounds. Multiple independent lines were examined. To control for potential variation in copy number between the *dex-1p*(IRS∆)::*gfp* strain and *dex-1p*::*gfp*, we examined multiple independent lines. The data were analyzed using one-way ANOVA and Bonferroni’s multiple comparisons test. Ten animals were measured for each genotype.

Mosaic analysis was conducted using *dex-1(ns42)* dauer animals expressing extrachromosomal *dex-1p*::*dex-1* and *sur-5*::*gfp* (Yochem *et al.* 1998). Lateral view micrographs were taken of transgenic animals and seam cells were scored for the presence of *sur-5*::*gfp* in the nucleus. Areas of lateral alae adjacent to seam cell nuclei positive for *sur-5*::*gfp* expression were scored as either full, partial, or no rescue. Seam cells not expressing *sur-5*::*gfp* were also counted and the adjacent alae scored. Twelve animals were observed for mosaicism, with one set of seam cells being scored for each animal.

### Data availability statement

All strains and plasmids are available upon request. Table S1 contains all plasmid constructs used in this study. Figure S1 shows the *dex-1(ns42)* body diameter data for dauers and L3s. Figure S2 shows the results of fluorescent bead feeding and pharyngeal pumping assays. Figure S3 shows that dauer-specific *lag-2p*::*gfp* is present in *dex-1(ns42)* dauers. Figure S4 shows that starved and pheromone-induced dauers are morphologically similar. Figure S5 describes *dyf-7(m537)* dauer morphology. Figure S6 contains a dose-response curve of SDS resistance of wild type and single- and double-mutants. Figure S7 contains gene schematics of cuticlin (CUT) mutants used in this study. Supplemental material available at Figshare: https://doi.org/10.25386/genetics.7077146.

## Results

### DEX-1 is required for proper dauer morphology and behavior

**Figure 2:**
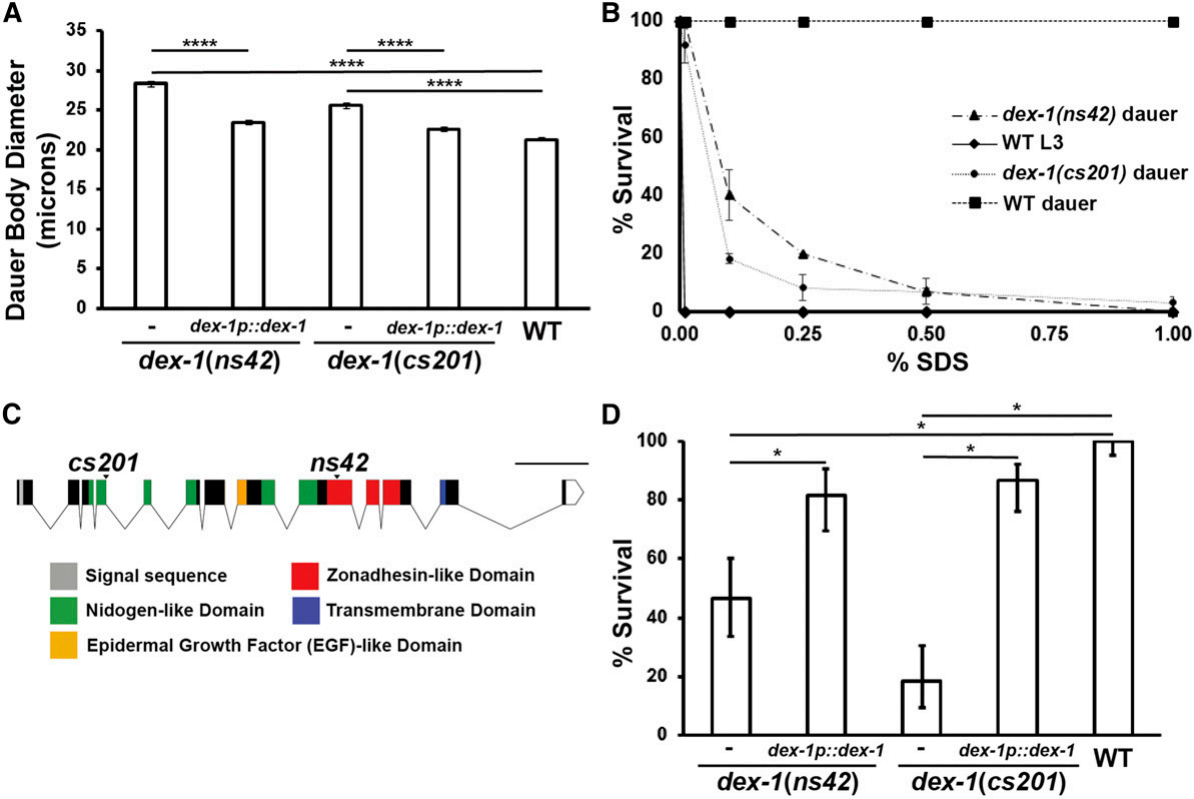
*dex-1* mutants form partial dauers. (A) *dex-1* mutant dauers are defective for dauer-specific radial shrinkage. The radial shrinkage defect can be rescued with *dex-1* cDNA driven by its endogenous promoter (*n* = 60 per genotype pooled from three trials). **** indicates statistical significance at *P* < 0.0001. Error bars indicate SEM. (B) Dose-response survival assay to SDS. *dex-1* dauers are sensitive to SDS compared to wild-type dauers, but are able to survive low levels of SDS exposure. Nondauer animals are sensitive at all tested SDS concentrations (*n* = 60 per treatment and dose pooled from three independent trials). Error bars indicate SEM from three independent trials. (C) *dex-1* gene schematic. DEX-1 contains two nidogen-like domains, a single epidermal growth factor-like domain, a low-complexity domain previously predicted to have similarity to zonadhesin, and a transmembrane domain. The *cs201* mutant allele is a point mutation at the exon 4 splice donor. The previously isolated *ns42* mutation truncates the DEX-1 protein in its predicted zonadhesin-like domain. Bar, 1 kb. (D) Percent survival of dauers at 0.1% SDS. The *dex-1* SDS sensitivity phenotype is partially rescued by the endogenous *dex-1* promoter and DEX-1 cDNA (pMH7) (*n* = 60 per genotype pooled from three independent trials). Error bars indicate 95% confidence intervals. Nonoverlapping confidence intervals were considered significantly different (*).

Wild-type *C. elegans* dauers have a distinctive morphology due to radial shrinkage that leads to a thin appearance compared with nondauers (Figure 1, A and B). We found that *dex-1(ns42)* mutants produce dauers that are defective in radial shrinkage, leading to a “dumpy dauer” phenotype (Figures 1C and 2A). The defect in body size appears specific to the dauer stage, as comparable nondauer *dex-1(ns42)* mutant L3s show no differences in body size compared with wild-type L3s (Figure S1). Radial shrinkage in dauers is correlated with the formation of longitudinal cuticular ridges on the lateral sides of the animal, called the alae (Cassada and Russell 1975). The lateral alae of *dex-1(ns42)* mutant dauers are indistinct compared with wild-type dauers (Figure 1, C, E, and F).

In addition to radial shrinkage, dauers have an ability to survive high concentrations of SDS (Cassada and Russell 1975; Androwski *et al.* 2017). Wild-type dauers survive for hours in 1% SDS (Cassada and Russell 1975). We found that while *dex-1(ns42)* mutant dauers were able to survive significantly higher concentrations of SDS than wild-type nondauer animals, they were sensitive to 1% SDS (Figure 2B and Table 1).

**Table 1:** % SDS concentration necessary to kill 50% of animals tested for each genotype

Strain	LD_50_*^a^*
Wild-type dauer	>10% ± —
Wild-type L3	<0.01 ± —
*dex-1(ns42)*	0.15 ± 0.03
*cut-1(tm1126)*	0.28 ± 0.05
*cut-6(ok1919)*	2.02 ± 0.15
*cut-5(ok2005)*	0.25 ± 0.06
*cut-1*; *dex-1*	0.20 ± 0.03
*dex-1 cut-6*	0.71 ± 0.10
*dex-1*; *cut-5*	—^b^
*cut-1*; *cut-5*	0.08 ± 0.02
*cut-1*; *cut-6*	1.85 ± 0.10
*cut-6*; *cut-5*	0.01 ± —^c^

In all cases “-” indicates no variation. For WT dauer and L3 the LD50 was above or below the limits of the doses tested, respectively. For b (dex-1; cut-5), the LD50 was not determined due to the early larval lethality.

For c, (cut-6; cut-5) survival was exactly 50% at the lowest dose (0.01%) and there were no survivors at higher concentrations. Thus, no 95% CI is available using the Probit analysis.

a 50% lethal dose ± 95% confidence interval as determined by probit analysis. Genotypes with nonoverlapping confidence intervals are considered statistically different.

b Synthetic lethal at L1.

c Synthetic dumpy at all stages.

The previously isolated *dex-1(ns42)* allele results in a premature stop codon in exon 9, encoding the predicted zonadhesin-like functional domain (Heiman and Shaham 2009) (Figure 2C). In an unrelated screen for mutants with embryonic and early larval lethality, we isolated *dex-1(cs201)*, which introduces a point mutation at the splice donor site of intron 4 (Figure 2C; also see Cohen *et al.* (2018). Analysis of *dex-1(cs201)* cDNA suggests this results in multiple transcripts with read through into intron 4, with all introducing a new stop codon 10 bp into this intron (Cohen *et al.* 2018). *dex-1(cs201)* mutants have a high rate of early larval lethality (96% dead L1s, *n* = 170). Similar to *dex-1(ns42)*, *dex-1(cs201)* dauers are defective in radial shrinkage, alae formation, and SDS resistance (Figures 1D and 2, A and B). To confirm *dex-1* as regulating these phenotypes, we rescued the radial shrinkage phenotype and SDS sensitivity of both *dex-1* mutants with *dex-1* cDNA under the control of its endogenous promoter (Figure 2, A and D). Together, these data suggest that *dex-1* mutants form partial dauers with defects in epidermal remodeling. The enhanced sensitivity of *dex-1(cs201)* to SDS compared with *dex-1(ns42)* (Figure 2D) combined with our molecular data suggest that *dex-1(ns42)* is a hypomorphic allele; however, due to the early larval lethality of *dex-1(cs201)*, all further experiments were done using the *dex-1(ns42)* background, unless otherwise noted.

We characterized additional dauer-specific phenotypes in *dex-1(ns42)* mutants. Dauers suppress pharyngeal pumping and have a buccal plug that prevents feeding (Cassada and Russell 1975; Popham and Webster 1979). Nondauers will readily ingest fluorescent beads and show fluorescence throughout the digestive system (Nika *et al.* 2016). We did not find fluorescent beads in *dex-1(ns42)* mutant dauer intestines; however, we occasionally observed them in the buccal cavity (Figure S2, A–C). Additionally, we found no difference in the rate of pharyngeal pumping between *dex-1(ns42)* and wild-type dauers (Figure S2D). These data suggest that while pharyngeal pumping is efficiently suppressed, *dex-1(ns42)* dauers have low-penetrance defects in buccal plug formation. Finally, we examined *dex-1(ns42)* dauers for the presence of dauer-specific gene expression of *lag-2p*::*gfp* in the IL2 neurons during dauer (Ouellet *et al.* 2008). Similar to wild-type dauers, *dex-1(ns42)* dauers showed appropriate dauer-specific expression (Figure S3).

**Figure 3:**
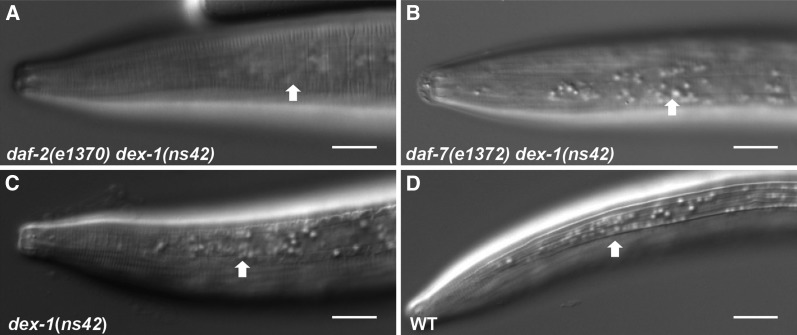
*dex-1* functions outside of the dauer decision pathway. (A–D) Lateral view micrographs of (A) *daf-2(e1370) dex-1(ns42)*, (B) *daf-7(e1372) dex-1(ns42)*, (C) *dex-1(ns42)*, and (D) wild-type dauers. *daf-c* mutations in the dauer decision pathway did not suppress the *dex-1(ns42)* alae phenotype. Arrows point to the lateral alae. Bar, 10 μm.

We sought to determine whether the *dex-1(ns42)* mutation would affect the initial decision to enter dauer. The dauer formation decision is based on the ratio of population density to food availability during early larval development (Golden and Riddle 1984). *C. elegans* constitutively secrete a pheromone mixture that is sensed by conspecific animals and, at high levels, triggers dauer formation (Golden and Riddle 1982). Dauers can be picked from old culture plates (starved) or can be induced using purified dauer pheromone. We found no difference in the dauer morphology phenotype between starved or pheromone-induced *dex-1(ns42)* mutant dauers (Figure S4). The *C. elegans* insulin/IGF-like and TGF-β signaling pathways function in parallel with regulate dauer formation (Thomas *et al.* 1993; Gottlieb and Ruvkun 1994). Reduced insulin and TGF-β signaling induced by overcrowding and scarce food promotes dauer formation (Riddle and Albert 1997). Disruption of either the insulin-receptor homolog DAF-2 or the TGF-β homolog DAF-7 results in a *daf-c* phenotype. The *dex-1(ns42)* alae formation defects were not suppressed in the *daf-7(e1372)* or *daf-2(e1370)* mutant backgrounds. While nonnull alleles were used in these crosses, these data may suggest that DEX-1 acts outside of the dauer formation decision to generate dauer alae (Figure 3).

### DEX-1 is secreted, but acts locally to regulate seam cell remodeling during dauer morphogenesis

**Figure 4:**
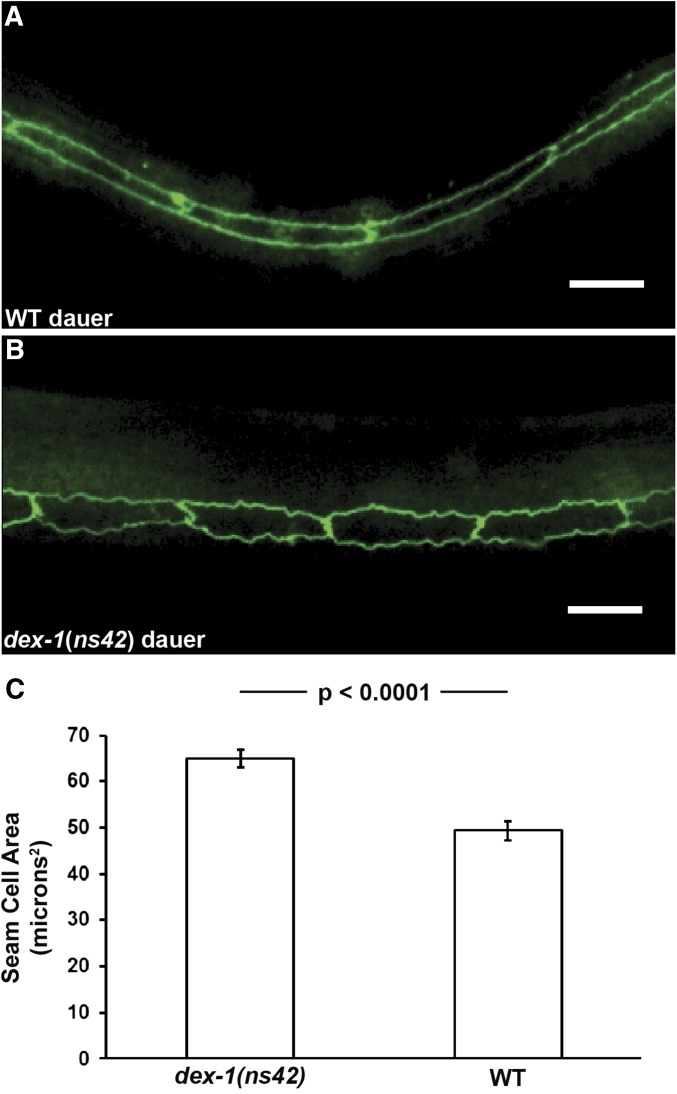
*dex-1* mutant dauers have defects in seam cell shrinkage. Lateral view micrographs of (A) wild-type (WT) and (B) *dex-1(ns42)* dauers expressing the apical junction marker *ncIs13* [*ajm-1*::*gfp*]. The seam cells of wild-type dauers are elongated and smooth, while *dex-1(ns42)* mutant seam cells are wider with jagged edges. Bar, 10 μm. (C) Quantification of seam cell area as measured with the *ajm-1*::*gfp* reporter. Data were analyzed by an unpaired *t*-test (*dex-1(ns42)* dauer *n* = 14, WT dauer *n* = 15). Error bars indicate SEM.

Dauer-specific radial shrinkage and subsequent lateral alae formation are facilitated by shrinkage of the seam cells (Singh and Sulston 1978; Melendez *et al.* 2003). Using the *ajm-1*::*gfp* apical junction marker (Köppen *et al.* 2001), we found that *dex-1(ns42)* mutant dauer seam cells are larger and have jagged, rectangular edges, unlike the smooth, elongated seam cells of wild-type dauers (Figure 4). These data suggest that DEX-1 is required for seam cell remodeling.

**Figure 5:**
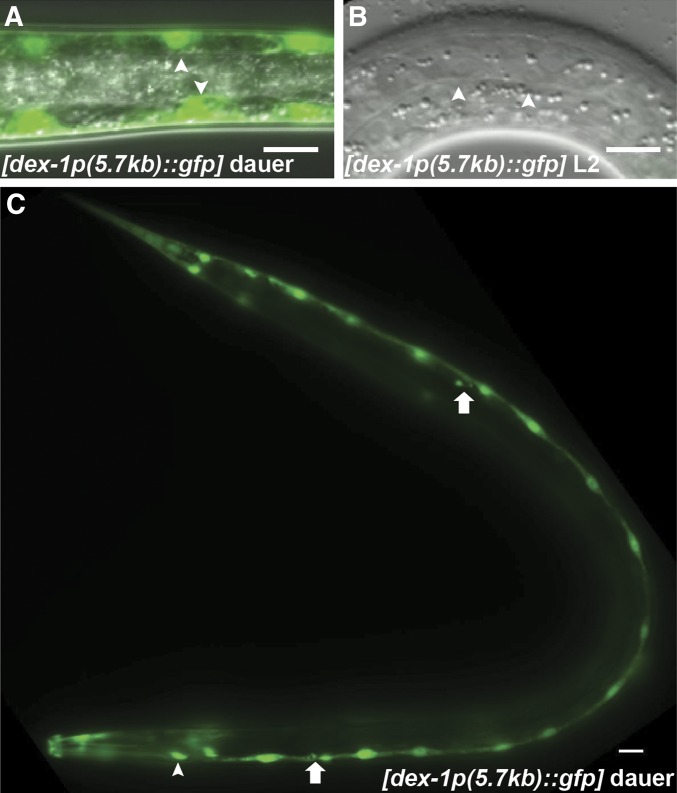
*dex-1* is expressed in the seam cells during dauer. (A) Dorsoventral view of a dauer animal expressing *dex-1p*::*gfp* from a 5.7 kb promoter. (B) GFP is seen in the seam cells (arrowheads) during dauer and is not expressed during L3. Bar, 10 μm. (C) Lateral view of a dauer animal expressing *dex-1p*::*gfp* from a 5.7 kb *dex-1* promoter region. In addition to the seam cells along the length of the animal, *dex-1* is expressed in the socket glial cells of the anterior and posterior deirid neurons (arrows) during dauer, and in unidentified pharyngeal cells (arrowhead) during all larval stages. Bar, 10 μm.

To determine the location of *dex-1* expression, we generated transgenic animals expressing green fluorescent protein (GFP) driven by a 5.7 kb 5′ *dex-1* upstream promoter. We observed bright fluorescence in the seam cells and glia socket cells of the anterior and posterior deirid neurons starting in the predauer L2 (L2d) stage. Expression of *dex-1p*::*gfp* in the seam cells and deirid socket cells persisted throughout dauer (Figure 5). We also observed *dex-1p*::*gfp* expression in unidentified pharyngeal cells during all larval stages (Figure 5).

**Figure 6:**
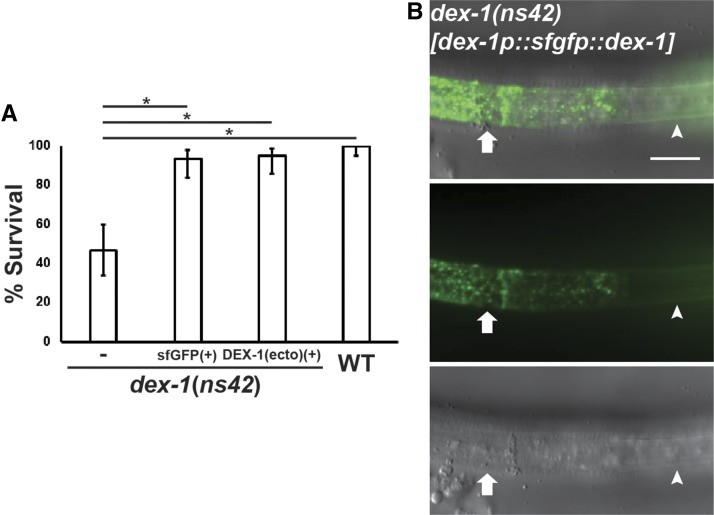
DEX-1 is localized to the outer ridges of the lateral alae. (A) Functional full-length *dex-1p*::*sfgfp*::*dex-1* and truncated *dex-1p*::*dex-1(ecto)*::*sfgfp* constructs rescue the *dex-1(ns42)* SDS sensitivity phenotype to wild-type levels. Error bars indicate 95% confidence intervals. Nonoverlapping confidence intervals were considered significantly different (*) (*n* = 60 per genotype pooled from three independent trials). (B) Lateral view overlay (top), fluorescence (middle), and DIC (bottom) micrograph of a *dex-1(ns42)* dauer expressing a full-length *dex-1p*::*sfgfp*::*dex-1* construct. The full-length *dex-1p*::*sfgfp*::*dex-1* construct rescues the lateral alae phenotype in a mosaic pattern. *dex-1*::*sfgfp*::*dex-1* localizes in a diffuse pattern to the areas immediately below the outer ridges of rescued, intact lateral alae (arrowheads). In contrast, in areas where lateral alae are not rescued, DEX-1 expression is bright and punctate and localizes throughout the lateral ridge (arrows). Bar, 10 μm.

To determine the subcellular localization of DEX-1 during dauer remodeling, we expressed *dex-1* cDNA (isoform A) tagged with super-folder GFP (sfGFP) under the control of its endogenous promoter. This *dex-1p*::*sfgfp*::*dex-1* construct rescued the SDS phenotype in *dex-1(ns42)* mutant dauers, suggesting it is functional (Figure 6A). During dauer, we observed sfGFP along the length of the animal above seam cells in a mosaic pattern alternating between alae with diffuse sfGFP expression immediately under the outer ridges and alae with bright and punctate expression immediately below the lateral ridge (Figure 6B). Interestingly, regions with diffuse GFP expression correlated with proper radial constriction and intact lateral alae, whereas regions with bright, punctate expression did not undergo proper dauer shrinkage and lacked alae (Figure 6B). The mosaicism in alae formation was observed in both wild-type and *dex-1(ns42)* dauers expressing the *dex-1p*::*sfgfp*::*dex-1* construct, indicative of dominant negative effects of the transgene.

**Figure 7:**
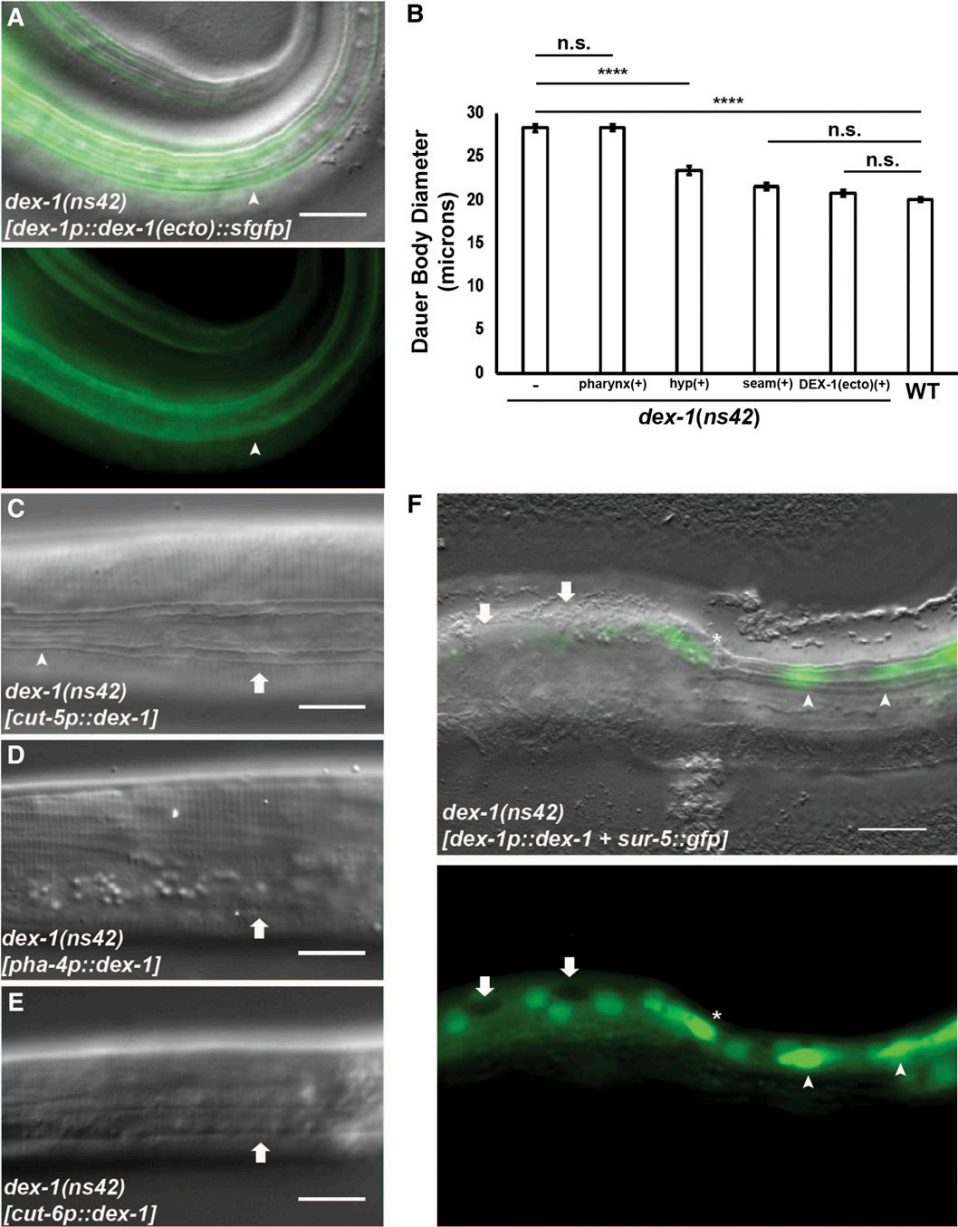
DEX-1 functions as a secreted protein in a tightly localized manner. (A) Lateral view DIC overlay (top) and fluorescence (bottom) images of a *dex-1(ns42)* dauer expressing *dex-1p*::*dex-1(ecto)*::*sfgfp*, which lacks the C-terminal transmembrane domain. *dex-1p*::*dex-1(ecto)*::*sfgfp* localizes to the outer edges of the lateral ridge and completely rescues the *dex-1(ns42)* alae phenotype. Arrowheads point to areas with distinct lateral alae. (B) Cell-specific rescue of body diameter in *dex-1(ns42)* suggests DEX-1 functions cell-autonomously. Error bars indicate SEM. **** indicates statistical significance at *P* < 0.0001 (*n* = 15). (C) *dex-1* expression from *the cut-5* seam cell-specific promoter rescues the *dex-1(ns42)* alae formation phenotype in a mosaic pattern. Arrowhead points to areas with rescued alae, arrow points to areas with partially rescued alae. *dex-1* expression from a (D) *pha-4* pharyngeal and (E) *cut-6* hypodermal promoter fail to rescue alae formation in *dex-1(ns42)* dauers. Arrows point to the indistinct lateral alae. (F) DIC overlay (top) and fluorescence (bottom) image of a *dex-1(ns42)* dauer expressing a *dex-1p*::*dex-1* construct with *sur-5*::*gfp. sur-5*::*gfp* expression in seam cell nuclei was correlated with rescue of the lateral alae (arrowheads), while the absence of *sur-5*::*gfp* correlated with indistinct lateral alae and a larger body diameter (arrows). Occasionally, *sur-5*::*gfp* was expressed in seam cell nuclei that did not show lateral alae rescue (*). Bar, 10 μm.

DEX-1 contains a putative transmembrane domain, but was previously suggested to be secreted through cleavage of the large extracellular domain (Heiman and Shaham 2009). To test if DEX-1 can function as a secreted protein during dauer, we expressed a *dex-1p*::*dex-1(ecto)*::*sfgfp* construct that truncates the C-terminal transmembrane domain. Consistent with a role as a secreted protein, the truncated DEX-1 construct rescued the SDS resistance (Figure 6A), radial shrinkage, and alae formation phenotypes (Figure 7, A and B).

To further examine where DEX-1 acts to regulate seam cell remodeling, we expressed *dex-1* cDNA under the control of cell-specific promoters. First, we expressed *dex-1* in the seam cells using the *cut-5* promoter. *cut-5* was previously shown to be expressed specifically in the seam cells during L1 and dauer (Sapio *et al.* 2005). Seam cell-specific expression of *dex-1* rescued the *dex-1(ns42)* radial shrinkage in a mosaic pattern similar to that seen with the full-length *sfgfp*::*dex-1* construct (Figure 7, B and C).

Expression of *dex-1* under a pharyngeal promoter failed to rescue the *dex-1* seam cell phenotype, suggesting that *dex-1* expression is necessary near the seam cells (Figure 7, B and D). The basolateral membranes of the seam cells are surrounded by a syncytial hypodermis. We hypothesized that the close proximity of the surrounding hypodermis to the seam cells would be sufficient for secreted DEX-1 to function during seam cell remodeling. We therefore expressed *dex-1* under the control of a *cut-6* promoter that was previously shown to drive expression in the hypodermis, but not in the seam cells, during dauer (Muriel *et al.* 2003). While *dex-1* expression in the surrounding hypodermis failed to rescue the *dex-1* dauer-specific alae phenotype (Figure 7E), hypodermal expression of *dex-1* resulted in partial rescue of the radial shrinkage phenotype, suggesting a limited ability for DEX-1 to translocate *in vivo* (Figure 7B).

Finally, to verify that DEX-1 is functioning in a cell-autonomous manner, we performed a mosaic analysis using *dex-1p*::*dex-1* and *sur-5*::*gfp* as the co-injection marker (Yochem *et al.* 1998). We found that in cells expressing *sur-5*::*gfp*: 56% showed full rescue of the lateral alae, 29% showed at least partial alae rescue, and 16% showed no alae rescue (*n* = 12 animals, 191 cells). We did not observe alae rescue in cells that did not express nuclear *sur-5*::*gfp* (Figure 7F). Together, these data indicate that *dex-1* acts cell-autonomously to regulate seam cell remodeling during dauer.

### DEX-1 regulates locomotion during dauer

**Figure 8:**
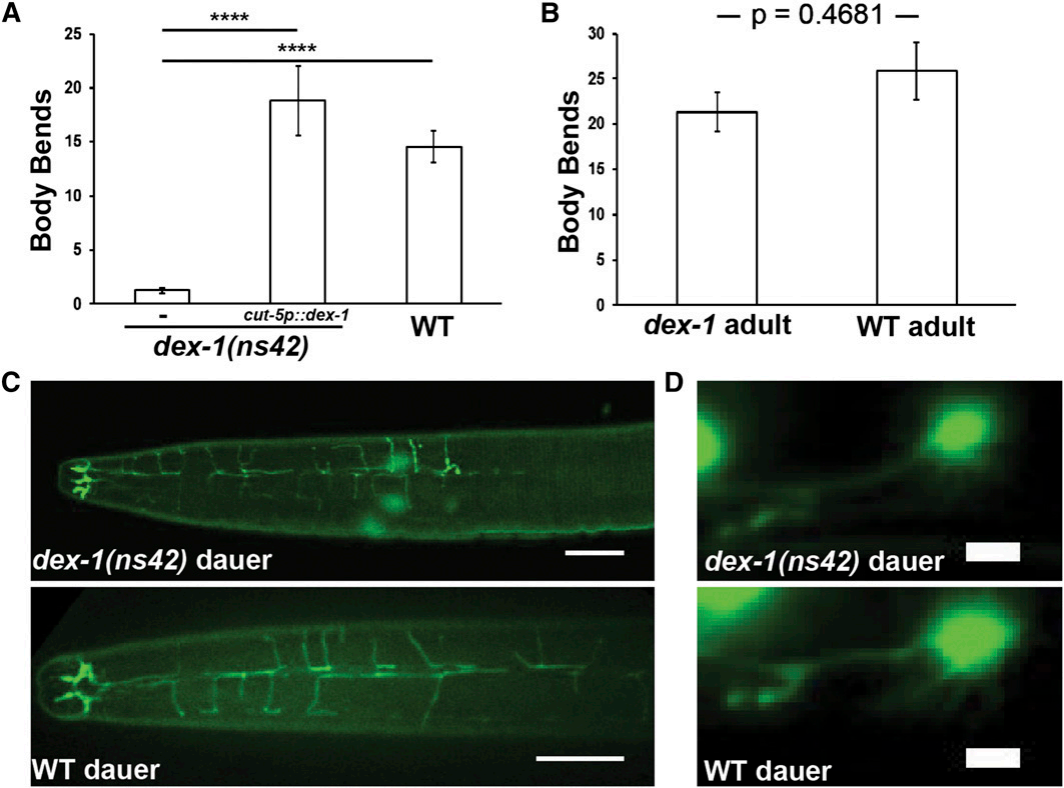
*dex-1(ns42)* dauers have defects in locomotion. (A) *dex-1(ns42)* dauers are less responsive when mechanically stimulated compared to wild-type dauers. Seam cell-specific expression of *dex-1* rescues the locomotion phenotype to wild-type levels. **** indicates statistical significance at *P* < 0.0001 (*n* = 40). (B) The locomotion defect in *dex-1(ns42)* dauers is dauer-specific, as nondauer *dex-1(ns42)* adults move at wild-type levels when mechanically stimulated (*n* = 40). Error bars indicate SEM. (C) Dorsoventral confocal images of the inner labial 2 (IL2) neurons in *dex-1(ns42)* (top) and wild-type (bottom) dauers. Bar, 10 μm. (D) Lateral view micrographs of the anterior deirid (ADE) neurons in *dex-1(ns42)* (top) and wild-type (bottom) dauers. We did not observe any structural differences in either the IL2 or deirid neurons. Bar, 1 μm.

Morphological changes during dauer are accompanied by changes in behavior. Wild-type dauer animals are often quiescent, but move rapidly when mechanically stimulated (Cassada and Russell 1975). Anecdotally, we noticed a higher percentage of quiescent *dex-1* dauers than wild-type dauers. To quantify this behavior, we developed a behavioral assay to measure movement following mechanical stimulation (see *Materials and Methods*). Although both *dex-1(ns42)* mutant and wild-type dauers initially respond to mechanical stimulation, the *dex-1* mutant dauers have significantly reduced locomotion and display slightly uncoordinated body movements (Figure 8A). This locomotion defect was dauer-specific, as nondauer *dex-1* animals moved at wild-type levels following mechanical stimulation (Figure 8B).

In addition to seam cell remodeling, the IL2 and deirid sensory neurons remodel during dauer formation (Albert and Riddle 1983; Schroeder *et al.* 2013). The IL2s regulate dauer-specific behaviors (Lee *et al.* 2011; Schroeder *et al.* 2013), while the deirids respond to specific mechanical cues (Sawin *et al.* 2000). We therefore examined these neuron classes using fluorescent reporters; however, we observed no obvious difference in neuronal structure between *dex-1(ns42)* and wild type (Figure 8, C and D). Given that *dex-1* was primarily expressed in the seam cells during dauer, we tested if seam cell-specific expression could rescue the behavioral phenotype. Surprisingly, seam cell-specific *dex-1* expression completely rescued the *dex-1(ns42)* dauer locomotion defects (Figure 8A).

### *dex-1* expression in dauers is regulated by DAF-16

**Figure 9:**
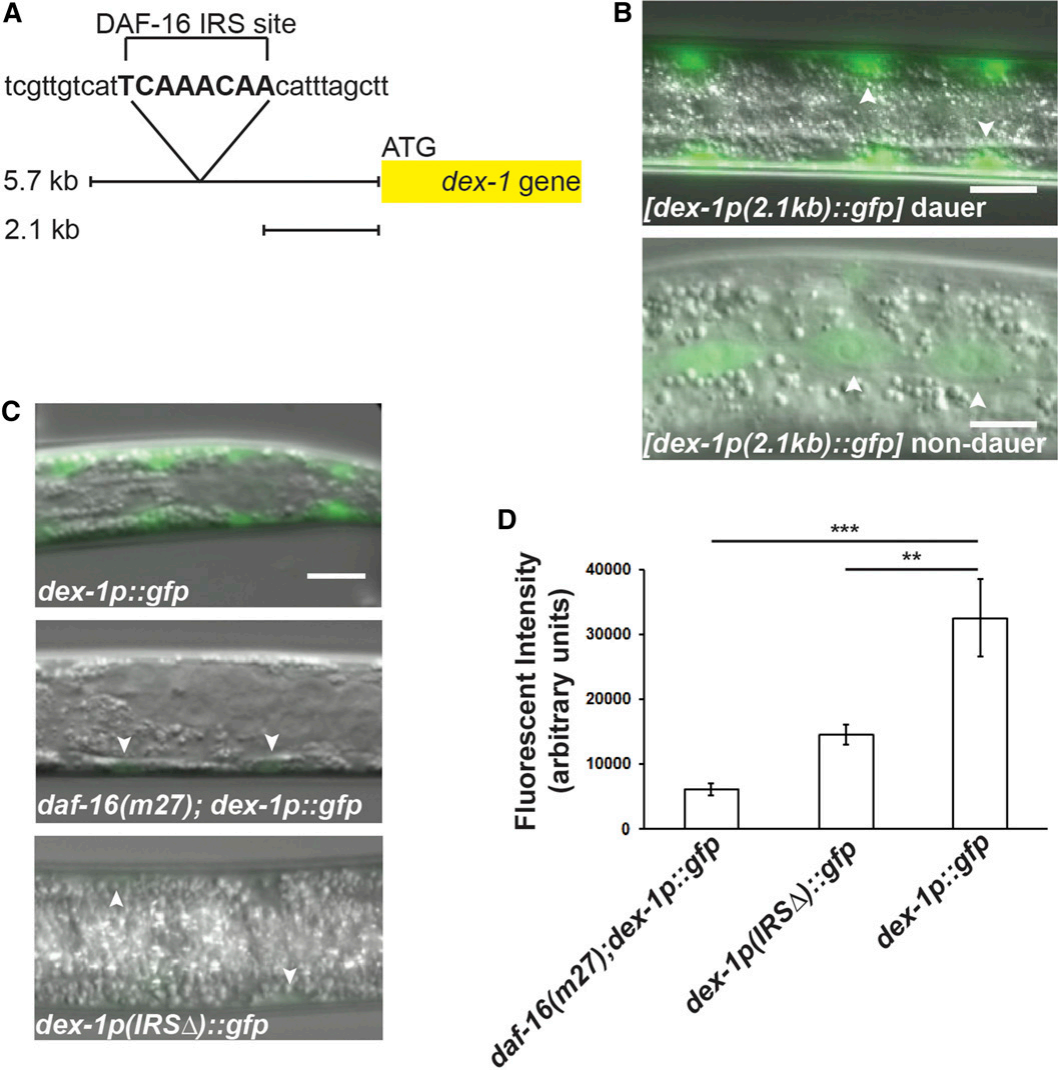
*dex-1* expression in the seam cells is regulated by DAF-16. (A) We identified a putative DAF-16 IRS binding site (in capitals) ∼3 kb upstream from the *dex-1* ATG start site. (B) Expression of *dex-1p*::*gfp* from a truncated 2.1 kb *dex-1* promoter drives fluorescence in the seam cells (arrowheads) in both dauer (top) and nondauer (bottom) stages. Bar, 10 μm. (C) Dorsoventral view micrographs of GFP expression from the 5.7 kb *dex-1* promoter in a wild-type background (top) produces bright fluorescence in the seam cells during dauer (also see Figure 5A). Fluorescence intensity is reduced in a *daf-16*(*m27)* partial dauer mutant background (middle). Deletion of the DAF-16 IRS sequence (bottom) from the 5.7 kb promoter region also significantly reduces GFP expression in the seam cells during dauer. Arrowheads indicate seam cells. Bar, 10 μm. (D) Quantification of GFP expression driven by the 5.7 kb *dex-1* promoter in dauers. ** and *** indicate statistical significance at *P* < 0.01 and *P* < 0.001, respectively. Error bars indicate SEM.

To understand how *dex-1* expression is regulated, we examined the 5′ upstream region of *dex-1* for potential regulatory sites. Previous chromatin immunoprecipitation sequencing data identified a putative DAF-16 binding site ∼3 kb upstream of the *dex-1* coding region (Figure 9A) (Celniker *et al.* 2009). DAF-16 is the sole *C. elegans* ortholog of the human Forkhead Box O-type transcription factor and a major regulator of the dauer decision (Lin *et al.* 1997; Ogg *et al.* 1997). To examine whether this region affects expression of *dex-1*, we first expressed GFP from a truncated 2.1 kb *dex-1* promoter that does not include the putative DAF-16 binding site. Unlike the 5.7 kb *dex-1p*::*gfp* promoter fusion, which resulted in GFP expression in the seam cells exclusively during dauer (Figure 5, A and B), the shorter *dex-1* promoter drove GFP expression in the seam cells during all larval stages (Figure 9B). This suggests that *dex-1* is repressed during nondauer stages and activated by DAF-16 during dauer.

To further examine if DAF-16 is regulating *dex-1* expression during dauer, we examined the expression of the 5.7 kb *dex-1p*::*gfp* reporter in a *daf-16(m27)* mutant background. While mutations in *daf-16* result in animals incapable of forming dauers, under high-pheromone concentrations, *daf-16(m27)* mutants can enter into a partial dauer state with some dauer morphological characteristics (Vowels and Thomas 1992; Gottlieb and Ruvkun 1994). *daf-16(m27)* partial dauers are identifiable by body morphology and the presence of indistinct lateral alae (Vowels and Thomas 1992). We found that the fluorescence intensity of *dex-1p*::*gfp* was significantly reduced in *daf-16(m27)* partial dauers compared to wild type, suggesting that DAF-16 regulates *dex-1* seam cell expression during dauer (Figure 9, C and D).

FOXO/DAF-16 binds to canonical DAF-16 binding elements and insulin response sequences (IRS) (Paradis and Ruvkun 1998; Obsil and Obsilova 2008). Within the chromatin immunoprecipitation sequencing–identified region (Celniker *et al.* 2009), we identified a putative IRS binding site (Figure 9A). To determine whether the identified DAF-16 IRS site directly regulates *dex-1* expression, we deleted the IRS sequence in the 5.7 kb *dex-1* promoter region used to drive GFP. We found that deleting the IRS sequence results in reduced GFP expression in the seam cells during dauer, similar to the levels observed in *daf-16* partial dauers (Figure 9, C and D). Taken together, these results indicate the 2.1-kb region proximal to the *dex-1* start codon drives expression in the seam cells, and that an unidentified element within the 3.6-kb region upstream from the 2.1-kb activation site represses expression outside of dauer. This repression is counteracted by DAF-16 binding to the IRS during dauer formation.

### *dex-1* interacts with genes encoding ZP-domain proteins

DEX-1 acts with the ZP-domain protein DYF-7 to regulate primary dendrite extension during embryogenesis (Heiman and Shaham 2009). We therefore examined the *dyf-7(m537)* mutant for defects in dauer morphogenesis. Unlike *dex-1* mutants, *dyf-7* mutants are unable to enter dauer under typical dauer-inducing environmental conditions (Starich *et al.* 1995), and so we examined *dyf-7* mutants in a *daf-c* mutant background. *daf-7*; *dyf-7* double mutants had normal dauer-specific radial shrinkage and IL2 dendrite arborization (Figure S5).

**Figure 10:**
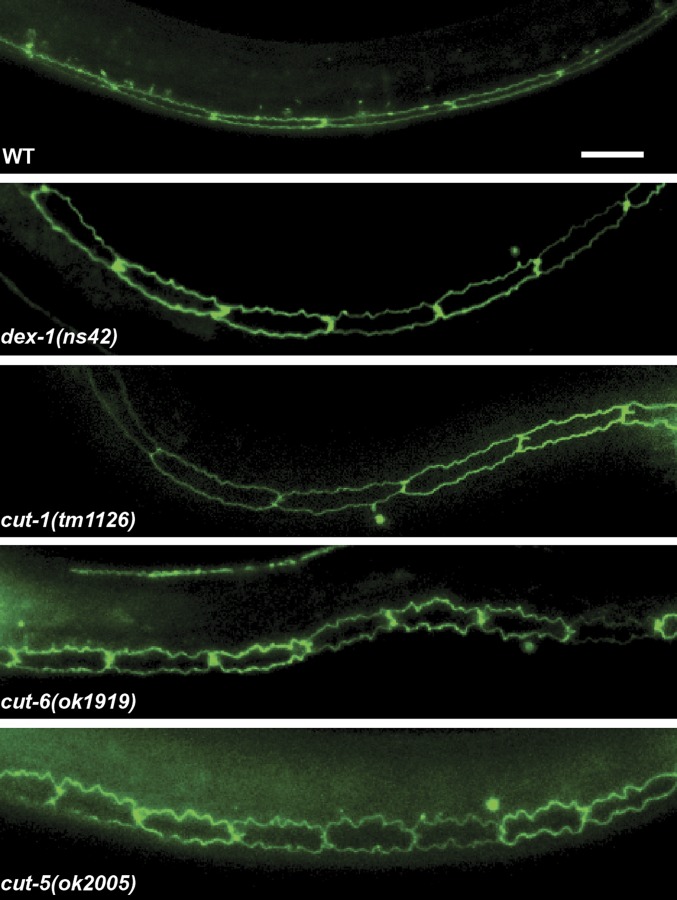
Cuticlin mutants phenocopy the *dex-1* mutant seam cell phenotype during dauer. Lateral view of wild-type, *dex-1(ns42)*, and cuticlin mutant dauers expressing the apical junction marker *ajm-1*::*gfp*. The seam cells in cuticlin mutants are jagged and wider, closely resembling those of *dex-1(ns42)* mutant dauers. Bar, 10 μm.

The cuticlin (CUT) proteins are a family of ZP-domain proteins originally isolated from nematode cuticles (Sebastiano *et al.* 1991). *cut-1* and *cut-5* are expressed in the seam cells, while *cut-6* is expressed in the surrounding hypodermis (Muriel *et al.* 2003; Sapio *et al.* 2005). Similar to *dex-1*, disruption of *cut-1*, *cut-5*, and *cut-6* results in dauers with incomplete radial shrinkage and defective alae formation (Sebastiano *et al.* 1991; Muriel *et al.* 2003; Sapio *et al.* 2005). We asked whether these defects in CUT mutant larvae were due to seam cell remodeling. We found that, similar to *dex-1(ns42)*, the seam cells of the CUT mutant dauers were enlarged with jagged edges (Figure 10). Also similar to *dex-1(ns42)* mutants, we found that *cut-1(tm1126)* and *cut-5(ok2005)* dauers were more sensitive to SDS compared with wild-type dauers (Figure S6 and Table 1). Interestingly, while *cut-6(ok1919)* mutant dauers were resistant to the standard 1% SDS treatment (Muriel *et al.* 2003), we found that the *cut-6* mutant dauers were substantially more sensitive to SDS than wild-type dauers (Figure S6 and Table 1). Taken together, these data indicate similar roles for DEX-1 and CUTs during dauer remodeling.

We hypothesized that, similar to its interaction with DYF-7 during embryogenesis, DEX-1 may genetically interact with the CUT proteins during dauer. We therefore examined double mutants of *dex-1(ns42)* with *cut-1(tm1126)*, *cut-5(ok2005)*, and *cut-6(ok1919)*. It is worth noting that, while the *cut* mutations are all deletion alleles (Figure S7), these data should be interpreted with caution as *dex-1(ns42)* is a hypomorphic allele. The *dex-1*; *cut-1* double mutant did not enhance SDS sensitivity beyond the *dex-1* single mutant, suggesting that they may act in the same pathway to regulate dauer remodeling (Figure S6 and Table 1). The *dex-1*; *cut-5* double mutant was synthetically lethal during embryogenesis or early L1. This is similar to the *dex-1(ns42)*; *dyf-7* double mutant (Heiman and Shaham 2009) and the severe loss-of-function mutant *dex-1(cs201)* (Cohen *et al.* 2018), suggesting that in addition to roles in dauer remodeling, CUT-5 has additional roles during early development. Interestingly, the *dex-1cut-6* double mutant was intermediate in SDS sensitivity between the *dex-1* and *cut-6* single mutants (Figure S6 and Table 1).

We further tested the *cut* mutant phenotypes by generating double mutants between each of the *cut* mutants (Figure S6 and Table 1). The *cut-1*; *cut-5* double mutant showed a significant reduction of SDS resistance compared to single mutants alone. Interestingly, the *cut-1*; *cut-6* double mutants retained the *cut-6* SDS sensitivity phenotype. The *cut-6*; *cut-5* dauers showed a drastic increase in sensitivity to SDS compared to single mutants. In addition, the *cut-6*; *cut*-5 double mutant showed a severe dumpy phenotype in all developmental stages. These results suggest that CUT-5 and CUT-6, like DEX-1 (Cohen *et al.* 2018), also play broader roles during development.

## Discussion

The dauer stage of *C. elegans* is an excellent example of a polyphenism, where distinct phenotypes are produced by the same genotype via environmental regulation (Simpson *et al.* 2011). Compared to the decision to enter dauer, little is known about the molecular mechanisms controlling remodeling of dauer morphology. DEX-1 was previously characterized for its role in embryonic neuronal development (Heiman and Shaham 2009), and in the accompanying article we show that it shapes multiple embryonic epithelia (Cohen *et al.* 2018). Our data show that DEX-1 also functions during dauer-specific remodeling of the stem cell-like seam cells.

We demonstrate that DEX-1 is secreted, but acts locally in a cell-autonomous manner to regulate seam cell remodeling during dauer morphogenesis. DEX-1 is similar in sequence to the human ECM protein SNED1 (Sushi, Nidogen, EGF-like domains 1). High levels of SNED1 expression promote invasiveness during breast cancer metastasis (Naba *et al.* 2014), suggesting a possible mechanical role in tissue remodeling. Interestingly, results from our full-length translational DEX-1 reporter indicate that seam cell remodeling can also be perturbed by a dominant negative effect of DEX-1 overexpression. Seam cells with bright, aggregated sfGFP::DEX-1 expression were correlated with indistinct lateral alae and large body diameter, whereas diffuse sfGFP::DEX-1 correlated with intact alae. We hypothesize that this could be the result of increased interaction of DEX-1 protein with either itself or other ZP proteins in the ECM, leading to protein aggregates that disrupt alae formation.

We also found the *dex-1* mutant dauers have defects in locomotion when mechanically stimulated. We originally assumed that this could be due to the lack of lateral alae; however, our seam cell-specific rescue of *dex-1* resulted in a mosaic pattern of alae formation while completely rescuing the behavior. One explanation may be that DEX-1 acts cell-nonautonomously to mediate dauer behaviors. Alternatively, our seam cell-specific promoter could drive undetectable expression in the neuromuscular system. Previous RNA interference data showed that knockdown of *dex-1* results in low penetrance defects in motor neuron commissure formation (Schmitz *et al.* 2007). Dauers also exhibit changes in somatic muscle structure (Dixon *et al.* 2008). We therefore speculate that *dex-1* mutant dauers may have defects in neuromuscular structure or function that results in locomotion defects.

**Figure 11:**
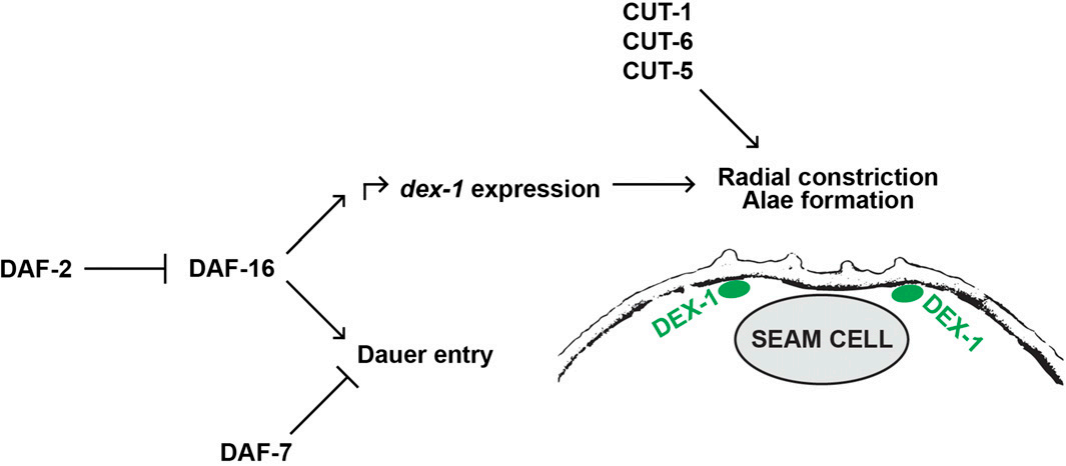
DEX-1 may function outside of the dauer decision pathway to facilitate dauer alae formation. A model diagram showing our proposed genetic pathway for *dex-1* transcriptional regulation. We show that during dauer, *dex-1(ns42)* is not suppressed by *daf-c* mutants and that *dex-1* is transcriptionally activated by DAF-16. We also show that DEX-1 functions as a secreted protein localized to the apical extracellular matrix that, along with the cuticlin proteins, facilitates dauer-specific radial constriction and alae formation.

Dissection of the genetic pathways regulating the decision to enter dauer has revealed insights into TGF-β, insulin, and hormone signaling (Thomas *et al.* 1993; Gottlieb and Ruvkun 1994; Riddle and Albert 1997). The FOXO transcription factor DAF-16 is a well-known regulator of the dauer formation decision by acting downstream of the insulin/IGF-1 receptor DAF-2 (Gottlieb and Ruvkun 1994; Ogg *et al.* 1997). Dauer-inducing environmental conditions lead to a translocation of DAF-16 to the nucleus, where it activates dauer formation pathways (Lee *et al.* 2001; Fielenbach and Antebi 2008). We found that *dex-1* expression during dauer is regulated by DAF-16. Based on our results, we propose that DEX-1 is repressed during nondauer postembryonic stages and DAF-16 serves to activate *dex-1* expression via an upstream IRS (Figure 11).

Our data suggests that DEX-1 acts along with ZP-domain proteins to control dauer remodeling. While the *cut* mutants are all deletion alleles that disrupt the ZP domains and, therefore, likely functional nulls (Figure S7), the *dex-1(ns42)* allele is a non-sense mutation late in the coding region (Heiman and Shaham 2009). Our isolation of the larval lethal *dex-1(cs201)* allele suggests that *dex-1(ns42)* is hypomorphic. Therefore, our *dex-1* genetic interaction experiments should be interpreted with caution. In addition to this complication, our double-mutant experiments do not provide a clear interaction pathway. For example, our results suggest that deletion of *cut-6* abrogates loss of *cut-1*. One possible explanation is a compensatory mechanism in the ECM, where loss of one ECM protein leads to increased expression of other structural components. This was previously shown in cases of osteogenesis imperfecta, where mutations in type I collagen led to increases in levels of thrombospondin and fibronectin (Fedarko *et al.* 1995). Alternatively, while we did not observe any obvious defects in the dauer formation decision in any of the double mutants, it is possible that slight differences in the ability to form dauers bias our observations toward individuals with only mild remodeling defects.

It has been proposed that biochemical compaction of the CUTs in the extracellular space between the seam and the hypodermis causes radial constriction, and thus forms the lateral alae via a “CUT tether” (Sapio *et al.* 2005). We add to this by proposing that DEX-1 is another seam-specific epidermal matrix component that, along with ZP CUTs, facilitates apical constriction of the seam and formation of dauer alae (Figure 11). Our data indicate that DEX-1 acts in a cell-autonomous manner directly beneath the edges of the lateral alae. Although expression of *dex-1* in the hypodermis was sufficient to partially rescue dauer-specific radial shrinkage, hypodermal expression failed to rescue the lateral alae phenotype. We therefore propose DEX-1 may act as a secreted protein during dauer with restricted localization to the cuticle or ECM immediately above the apical membrane of the seam cells. During embryogenesis, DEX-1 is secreted and localized to the dendritic tips (Heiman and Shaham 2009). DEX-1 may serve to couple physical interactions between the remodeled cuticular ECM and seam cell shape. Failure of these tissues to properly compact and thicken due to a loss of DEX-1 could lead to an overall weakening of the cuticle, and thus result in the SDS sensitivity observed in *dex-1* mutant dauers. Previous research demonstrated a role for autophagy in dauer-specific seam cell remodeling (Melendez *et al.* 2003). It will be interesting to determine if dauer-specific changes to autophagy are influenced by DEX-1–mediated mechanical forces.
